# Association of the COVID-19 Pandemic With Prehospital Characteristics and Outcomes of Pediatric Patients With Out-of-Hospital Cardiac Arrest in Japan, 2005-2020

**DOI:** 10.1001/jamanetworkopen.2022.35401

**Published:** 2022-10-06

**Authors:** Ling Zha, Sanae Hosomi, Kosuke Kiyohara, Tomotaka Sobue, Tetsuhisa Kitamura

**Affiliations:** 1 Division of Environmental Medicine and Population Sciences, Department of Social Medicine, Graduate School of Medicine, Osaka University, Suita, Japan; 2 Department of Traumatology and Acute Critical Medicine, Graduate School of Medicine, Osaka University, Suita, Japan; 3 Department of Food Science, Faculty of Home Economics, Otsuma Women’s University, Tokyo, Japan

## Abstract

This cohort study investigates the association of the COVID-19 pandemic with prehospital characteristics and outcomes of pediatric patients with out-of-hospital-cardiac arrest in Japan.

## Introduction

Sudden cardiac arrest is a major public health concern and a leading cause of death. Several pre–COVID-19 and post–COVID-19 comparisons have found an association between the COVID-19 pandemic and worse adult out-of-hospital cardiac arrest (OHCA) outcomes^1,2,3,4,5^; however, to our knowledge, studies on pediatric OHCA outcomes during the COVID-19 pandemic are limited. This study investigated the association of the COVID-19 pandemic with prehospital characteristics and outcomes of pediatric patients with OHCA in Japan.

## Methods

The All-Japan Utstein Registry has been described previously^6^ and is described in the eMethods in the Supplement. This study was approved by the ethics committee of Osaka University Graduate School of Medicine, which waived the requirement for informed consent owing to the retrospective nature of the study and because personal identifiers were not included in the Utstein records. The study followed the Strengthening the Reporting of Observational Studies in Epidemiology (STROBE) reporting guideline.

Pediatric patients (aged ≤17 years) with OHCA who were registered in the database between January 1, 2005, and December 31, 2020, were included based on eligibility (see exclusion criteria in eMethods in the Supplement). The primary outcome measured was 1-month survival. The secondary outcome measures included prehospital return of spontaneous circulation and 1-month survival with favorable neurologic outcome (defined as Cerebral Performance Categories scale category 1 [good cerebral performance] or 2 [moderate cerebral disability]). The groups were compared using the χ^2^ test for categorical variables and the Wilcoxon Mann-Whitney test for continuous variables. A linear trend test was used to evaluate the annual trends. Univariable and multivariable logistic regressions, with a focus on patients from the pre–COVID-19 (2015-2019) and post–COVID-19 (2020) eras, evaluated the association of COVID-19 with each outcome and compared outcomes between the declared emergency (April 7 to May 25) and the nonemergency periods in 2020. Crude and adjusted odds ratios (ORs) and 95% CIs were calculated. All tests were 2-tailed, and *P* < .05 indicated statistical significance.

## Results

Among the 23 000 participants whose data were recorded between 2005 and 2020 and analyzed, the proportions of 1-month survival improved annually (7.7% in 2005 and 12.3% in 2020; *P* < .001 for trend) (Figure). In total, 7603 participants (4567 boys [60.1%]; mean [SD] age, 6.2 [6.7] years) were included in the comparison between the pre–COVID-19 pandemic and post–COVID-19 pandemic eras (2015-2019 vs 2020; Table). The proportion of patients who received chest compression–only cardiopulmonary resuscitation (CPR) increased from 47.6% (3064 of 6443) in 2015-2019 to 52.9% (614 of 1160) in 2020, whereas the proportion of patients who received conventional CPR with rescue breathing decreased from 14.2% (914 of 6443) in 2015-2019 to 10.9% (126 of 1160) in 2020. The median emergency medical service response time increased from 8 minutes (IQR, 7-10 minutes) in 2015-2019 to 9 minutes (IQR, 7-11 minutes) in 2020 (*P* < .001). In 2020, no patients received shocks administered by public-access automated external defibrillators (AEDs) during the declared emergency, whereas 3.2% of patients (33 of 1027) received shocks during the nonemergency period (*P* = .04). The 1-month survival rate was 13.2% (852 of 6443) in 2015-2019 and 12.3% (143 of 1160) in 2020, without significant differences before and after the pandemic (adjusted OR, 0.95; 95% CI, 0.77-1.17). In 2020, the 1-month survival rate was 12.4% (127 of 1027) during the nonemergency period and 12.0% (16 of 133) during the declared emergency period (crude OR, 0.97; 95% CI, 0.56-1.69).

**Figure.**
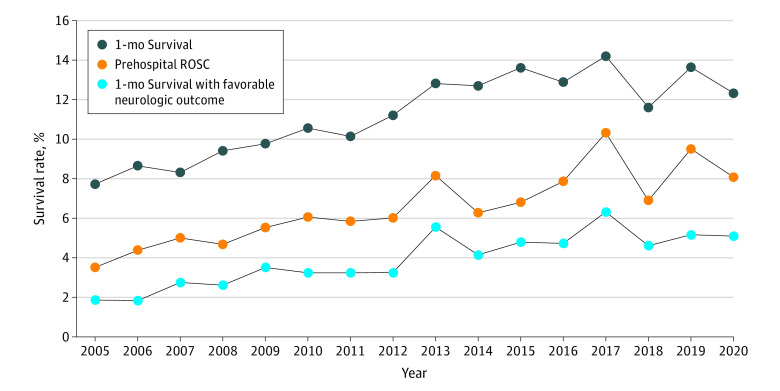
Annual Trend of Survival Outcomes From 2005 to 2020 Trend lines are based on an analysis of 23 000 patients aged 17 years or younger with out-of-hospital cardiac arrest. *P* values for trend were included in the analysis (all *P* < .001 for trend). ROSC indicates return of spontaneous circulation.

**Table.** Patient Characteristics and Survival Outcomes by Year

Characteristic or outcome	Patients, No. (%)			*P* value, 2015-2019 vs 2020	Patients, No. (%)		*P* value, nonemergency vs emergency periods
	Total (N = 7603)	2015-2019 (n = 6443)	2020 (n = 1160)		Nonemergency period (n = 1027)	Declared emergency period (April 7 to May 25) (n = 133)	
Patient characteristics							
Sex							
Female	3036 (39.9)	2547 (39.5)	489 (42.2)	.09	433 (42.2)	56 (42.1)	.99
Male	4567 (60.1)	3896 (60.5)	671 (57.8)		594 (57.8)	77 (57.9)	
Age group, y							
<1	2793 (36.7)	2385 (37.0)	408 (35.2)	.23	354 (34.5)	54 (40.6)	.16
1-17	4810 (63.3)	4058 (63.0)	752 (64.8)		673 (65.5)	79 (59.4)	
Type of bystander-witnessed status							
None	5449 (71.7)	4615 (71.6)	834 (71.9)	.79	733 (71.4)	101 (75.9)	.03
Family member	1265 (16.6)	1079 (16.7)	186 (16.0)		161 (15.7)	25 (18.8)	
Others	889 (11.7)	749 (11.6)	140 (12.1)		133 (13.0)	7 (5.3)	
Cardiac origin of arrest	2702 (35.5)	2285 (35.5)	417 (35.9)	.75	367 (35.7)	50 (37.6)	.67
Initial rhythm							
VF or pVT	444 (5.8)	377 (5.9)	67 (5.8)	.11	61 (5.9)	6 (4.5)	.77
PEA	1155 (15.2)	955 (14.8)	200 (17.2)		178 (17.3)	22 (16.5)	
Asystole	6004 (79.0)	5111 (79.3)	893 (77.0)		788 (76.7)	105 (78.9)	
Type of bystander-initiated CPR							
None	2885 (37.9)	2465 (38.3)	420 (36.2)	.001	366 (35.6)	54 (40.6)	.53
Chest compression–only CPR	3678 (48.4)	3064 (47.6)	614 (52.9)		549 (53.5)	65 (48.9)	
Conventional CPR with rescue breathing	1040 (13.7)	914 (14.2)	126 (10.9)		112 (10.9)	14 (10.5)	
Shocks by public-access AED	215 (2.8)	182 (2.8)	33 (2.8)	.97	33 (3.2)	0	.04
EMS response time, median (IQR), min	8 (7-10)	8 (7-10)	9 (7-11)	<.001	9 (7-11)	9 (8-11)	.68
Outcomes							
1-mo Survival	NA	852 (13.2)	143 (12.3)	NA	127 (12.4)	16 (12.0)	NA
Crude OR (95% CI)	NA	1 [Reference]	0.92 (0.76-1.12)	.41	1 [Reference]	0.97 (0.56-1.69)	.91
Adjusted OR (95% CI)^a^	NA	1 [Reference]	0.95 (0.77-1.17)	.60	1 [Reference]	Not available^b^	Not available^b^
Prehospital ROSC	NA	533 (8.3)	94 (8.1)	NA	85 (8.3)	9 (6.8)	NA
Crude OR (95% CI)	NA	1 [Reference]	0.98 (0.78-1.23)	.85	1 [Reference]	0.80 (0.39-1.64)	.55
Adjusted OR (95% CI)^a^	NA	1 [Reference]	0.99 (0.76-1.29)	.95	1 [Reference]	Not available^b^	Not available^b^
1-mo Survival with favorable neurologic outcome	NA	331 (5.1)	59 (5.1)	NA	52 (5.1)	7 (5.3)	NA
Crude OR (95% CI)	NA	1 [Reference]	0.99 (0.74-1.31)	.94	1 [Reference]	1.04 (0.46-2.34)	.92
Adjusted OR (95% CI)^a^	NA	1 [Reference]	1.04 (0.73-1.50)	.82	1 [Reference]	Not available^b^	Not available^b^

Abbreviations: AED, automated external defibrillator; CPR, cardiopulmonary resuscitation; EMS, emergency medical service; NA, not applicable; OR, odds ratio; PEA, pulseless electrical activity; pVT, pulseless ventricular tachycardia; ROSC, return of spontaneous circulation; VF, ventricular fibrillation.

^a^ Adjusted for sex, age group (<1 and 1-17 years), witness status (yes or no), cardiac or noncardiac origin, first-recorded rhythm (VF or pulseless VT, PEA or asystole), bystander CPR status (yes or no), and EMS response time (from call to patient contact).

^b^ Not available because of the limited number of cases owing to the declaration of a state of emergency in 2020.

## Discussion

In contrast to the decreased survival rate among adults with OHCA, our study revealed unchanged survival rates among pediatric patients with OHCA during the COVID-19 pandemic, suggesting that COVID-19 was not significantly associated with pediatric OHCA outcomes. The stable survival outcomes despite the longer emergency medical service response time and reduced rate of conventional CPR are potentially associated with the overall increase in bystander-initiated chest compressions in 2020. However, rates of conventional CPR with rescue breathing in 2020 and shocks administered using public-access AEDs during the declared emergency in 2020, which are 2 important prognostic factors associated with a high survival rate, decreased significantly. Efforts to strengthen bystander AED use, even during the COVID-19 pandemic, are needed to improve outcomes.^4^

A limitation of this study is that the Utstein style–based registry did not include information on the COVID-19 status of the individual patients. Nonetheless, our findings can help refine guidelines for health care resource management during the pandemic.

## Notes

Supplement.
eMethods.

eReferences.
