# Supplementary material for: Association of the COVID-19 Pandemic With Prehospital Characteristics and Outcomes of Pediatric Patients With Out-of-Hospital Cardiac Arrest in Japan, 2005-2020
Source: JAMA Netw Open. 2022 Oct 6;5(10):e2235401. doi: 10.1001/jamanetworkopen.2022.35401 (PMC9539716; doi:10.1001/jamanetworkopen.2022.35401)
Supplement: Supplement. — eMethods. eReferences. [file jamanetwopen-e2235401-s001.pdf]

Supplementary Online Content

Zha L, Hosomi S, Kiyohara K, Sobue T, Kitamura T. Association of the COVID-19 pandemic with prehospital characteristics and outcomes of pediatric patients with out-of-hospital cardiac arrest in Japan, 2005-2020. *JAMA Netw Open.* 2022;5(10):e2235401. doi:10.1001/jamanetworkopen.2022.35401

eMethods.
eReferences.

This supplementary material has been provided by the authors to give readers additional information about their work.

© 2022 Zha L et al. *JAMA Network Open.*

eMethods.

Study design and setting

The All-Japan Utstein Registry is a population-based prospective registry of out-of-hospital cardiac arrest (OHCA) based on the standardized Utstein style.[1,2] This observational study included pediatric patients with cardiac and non-cardiac OHCA who were resuscitated, whose arrests were witnessed by bystanders, and who were brought to medical facilities between January 1, 2005, and December 31, 2020. Adult patients with OHCA (age ≥18 years) were excluded because the characteristics and outcomes of OHCA vary between children and adults.[3,4] In Japan, the first case of coronavirus disease (COVID-19) was confirmed on January 15, 2020. A total of 239,192 COVID-19 patients were documented and 3,501 COVID-19-related deaths occurred until the end of 2020. There were three waves of the COVID-19 epidemic in Japan in 2020. From April 7 to May 25, 2020, a state of emergency was declared following the initial rapid spread of the COVID-19 infection and mortality.

Cardiac arrest is defined as the cessation of cardiac mechanical activity, which can be determined by the absence of signs of circulation.[5] The cardiac arrests recorded in this registry were categorized into cardiac and noncardiac origins, with the latter generally resulting from cerebrovascular disease, asphyxia, malignant tumors, external causes, drug overdose, anaphylaxis,

© 2022 Zha L et al. *JAMA Network Open.*

accidental hypothermia, and traffic accidents. The clinical diagnoses were made by the physician in charge along with the emergency medical service (EMS) personnel. A total of 27,221 pediatric OHCA patients (age 0–17 years) were registered between 2005 and 2020 in the All-Japan Utstein Registry. After excluding patients who were not resuscitated (N=1,596); arrest witnessed by EMS or unknown (N=1,707); and those with missing data for the first rhythm (N=876), outcomes (N=14), or bystander-initiated cardiopulmonary resuscitation (CPR; N=28), 23,000 participants were eligible for analysis. Furthermore, in the sub-analysis of differences between the pre-COVID-19 (2015–2019) and COVID-19 (2020) eras, 15,397 patients who were registered between 2005 and 2014 were excluded.

EMS organization in Japan

Detailed information on the EMS system in Japan has been reported elsewhere.[6] Briefly, the EMS system is operated by local fire stations. An ambulance from a nearby fire station is summoned when required, and 24/7 emergency services are available. Emergency life-saving technicians (ELST) comprise the majority of highly trained pre-hospital emergency care providers. An ambulance typically contains three emergency providers, including at least one ELST. OHCA patients can be provided an intravenous line, adjunct airway, and semiautomated external defibrillator. Specially trained ELSTs have been performing tracheal intubation and delivering

© 2022 Zha L et al. *JAMA Network Open.*

intravenous epinephrine since July 2004 and April 2006, respectively. Do-not-resuscitate orders and living wills are generally not accepted in Japan. EMS providers were not permitted to terminate resuscitation in the field. Consequently, the majority of OHCA patients who were treated by EMS personnel were transported to a hospital and enrolled in the All-Japan Utstein Project, with the exception of patients with decapitation, incineration, decomposition, rigor mortis, or dependent cyanosis.

Since July 2004, citizens have been legally permitted to use automated external defibrillators (AED). According to the Japanese guidelines for CPR, all EMS providers perform CPR.[7] Approximately 2 million citizens in Japan have participated in community CPR programs that provide training on chest compression, mouth-to-mouth ventilation, and AED use.[6,8] The telephone-assisted CPR by dispatchers was changed from conventional CPR to chest compression-only CPR in 2006, and dispatchers encourage bystanders to provide chest compression-only CPR if it is difficult for the bystanders to administer rescue breathing.[9] However, the EMS dispatcher instructions did not change with regard to the national recommendations in Japan from before to after the COVID-19 pandemic.[10] In addition to standard precautions, EMS personnel are required to wear N95 face masks and isolation gowns when attending to cardiac arrest patients during the COVID-19 pandemic. In accordance with the EMS protocol, since April 24, 2020, paramedics have been encouraged to use supraglottic airway

© 2022 Zha L et al. *JAMA Network Open.*

management instead of endotracheal intubation. Moreover, the fire departments ceased CPR training of the general public when a state of emergency was declared.

Data collection and quality control

Data were prospectively collected using a form that included the collection of the data that was recommended in the Utstein-style reporting guidelines for cardiac arrest.[1,2] These data points include patient's age, sex, type of bystander witness status, first-recorded cardiac rhythm, life support by EMS personnel (i.e., the use of advanced life support devices and insertion of an intravenous line), time course of resuscitation, epinephrine administration, prehospital return of spontaneous circulation (ROSC), and 1-month survival rates. Data on EMS call receipt time, arrival of the ambulance at the scene of the accident, time of contact with patients, initiation of CPR, time of defibrillation performed by EMS personnel, and arrival at the hospital were recorded using the clock of each EMS system. When bystanders delivered shocks using the public-access AED, the first-recorded rhythm was classified as ventricular fibrillation (VF) or pulseless ventricular tachycardia (VT). Information on the type of bystander CPR was obtained by EMS personnel through observation of and interviews with the bystander that were performed before leaving the scene of the accident. EMS personnel completed the data forms in cooperation with the treating physicians. Data were integrated into the All-Japan Utstein Registry database server

© 2022 Zha L et al. *JAMA Network Open.*

and logically checked using a computer system. When the data were incomplete, the Fire and Disaster Management Agency requested that the fire stations to supply missing details. However, the Utstein style-based registry neither provides data on the COVID-19 status of patients before the occurrence of arrests nor any information about in-hospital treatments.

All OHCA survivors were followed up by the EMS personnel in charge for up to 1 month after the incident. The 1-month survival with favorable neurologic outcome was determined by the attending physician using the Cerebral Performance Categories (CPC) scale as follows: Category 1, good cerebral performance; Category 2, moderate cerebral disability; Category 3, severe cerebral disability; Category 4, coma or vegetative state; and Category 5, death.[1,2]

Statistical analysis

Categorical variables are presented as numbers with percentages and were compared using the χ^2 test to identify intergroup differences (OHCA during or outside the state of emergency). Continuous variables are presented as the median with interquartile range (IQR), and intergroup comparisons were undertaken using the Wilcoxon Mann–Whitney test. A linear trend test was used to evaluate the annual trends. Both univariable and multivariable logistic regression analyses were used to identify the factors that were associated with 1-month survival, prehospital ROSC, and 1-month survival with favorable neurologic outcome. Crude and adjusted odds ratios (ORs)

© 2022 Zha L et al. *JAMA Network Open.*

and 95% confidence intervals (CIs) were calculated. In the multivariable analysis, biologically essential factors that were considered to be related to clinical outcomes were included as potential confounders,[6] and these variables included sex, age group (<1 and 1–17 years), witness status (yes or no), cardiac or noncardiac origin, first-recorded rhythm (VF or pulseless VT, pulseless electrical activity or asystole), bystander CPR status (yes or no), and EMS response time (from call to patient contact). All statistical analyses were conducted using STATA version MP 17.0 (StataCorp LP, College Station, TX). All tests were two-tailed, and $p<0.05$ indicated statistical significance.

Ethics statements

This study was approved by the ethics committee of Osaka University Graduate School of Medicine (approval number: 14147), and the information in the manuscript has been reported in compliance with the STROBE statement for reporting cohort and cross-sectional studies.[11] The requirement for written informed consent was waived owing to the retrospective nature of the study and because personal identifiers were not included in the Utstein records.

© 2022 Zha L et al. *JAMA Network Open.*

eReferences.

1. Cummins RO, Chamberlain DA, et al. Recommended guidelines for uniform reporting of data from out-of-hospital cardiac arrest: The Utstein style: A statement for health professionals from a task force of the American Heart Association, the European Resuscitation Council, the Heart and Stroke Foundation of Canada, and the Australian Resuscitation Council. *Circulation*. 1991;84(2):960-975.
2. Jacobs I, Nadkarni V, Bahr J, et al. Cardiac arrest and cardiopulmonary resuscitation outcome reports: update and simplification of the Utstein templates for resuscitation registries: a statement for healthcare professionals from a task force of the International Liaison Committee on Resuscitation (American Heart Association, European Resuscitation Council, Australian Resuscitation Council, New Zealand Resuscitation Council, Heart and Stroke Foundation of Canada, Inter-American Heart Foundation, Resuscitation Councils of Southern Africa). *Circulation*. 2004;110:3385-3397.
3. Nitta M, Iwami T, Kitamura T, et al. Age-specific differences in outcomes after out-of-hospital cardiac arrests. *Pediatrics*. 2011;128(4):e812-e820.
4. Atkins DL, Everson-Stewart S, et al. Epidemiology and out-of-hospital outcomes.
5. Kitamura T, Iwami T, Kawamura T, et al. Bystander-initiated rescue breathing for out-of-hospital cardiac arrest of non-cardiac origin. *Circulation*. 2010;122(3):293-299.
6. Kitamura T, Iwami T, Kawamura T, et al. Nationwide public-access defibrillation in Japan. *N Engl J Med*. 2010;362(11):994-1004.
7. Japan Resuscitation Council. *2010 Japanese Guidelines for Emergency Care and Cardiopulmonary Resuscitation*. 1st ed. Tokyo: Health Shuppansha; 2011 (in Japanese).
8. Fire and Disaster Management Agency of Japan. Effect of First Aid on Cardiopulmonary Arrest. https://www.fdma.go.jp/publication/rescue/post-2.html. Accessed May 3, 2022.

© 2022 Zha L et al. *JAMA Network Open.*

9. Iwami T, Kitamura T, Kiyohara K, Kawamura T. Dissemination of chest Compression–only cardiopulmonary resuscitation and survival after out-of-hospital cardiac arrest. *Circulation*. 2015;132(5):415-422.

10. Fire and Disaster Management Agency of Japan. Responses by Fire Departments for Cardiopulmonary Arrest Patients with COVID-19 Infection and Emergency Patients with Suspected COVID-19 Infection (in Japanese). https://www.fdma.go.jp/laws/tutatsu/items/200427_kyuki_1.pdf. Accessed August 1, 2022.

11. Gallo V, Egger M, McCormack V, et al. Strengthening the reporting of observational studies in epidemiology-molecular epidemiology STROBE-ME: An extension of the STROBE statement. *J Clin Epidemiol*. 2011;64(12):1350-1363.

© 2022 Zha L et al. *JAMA Network Open.*
